# NEFA‐induced ROS impaired insulin signalling through the JNK and p38MAPK pathways in non‐alcoholic steatohepatitis

**DOI:** 10.1111/jcmm.13617

**Published:** 2018-03-30

**Authors:** Wenwen Gao, Xiliang Du, Lin Lei, Heyuan Wang, Min Zhang, Zhe Wang, Xiaobing Li, Guowen Liu, Xinwei Li

**Affiliations:** ^1^ Key Laboratory of Zoonosis Ministry of Education College of Veterinary Medicine Jilin University Changchun Jilin Province China; ^2^ Department of Endocrinology and Metabolism The first Hospital Jilin University Changchun Jilin Province China

**Keywords:** insulin resistance, mitochondrial dysfunction, non‐alcoholic steatohepatitis, non‐esterified fatty acid, oxidative phosphorylation complex

## Abstract

The aim of this study was to investigate the changes in hepatic oxidative phosphorylation (OXPHOS) complexes (COs) in patients and cows with non‐alcoholic steatohepatitis (NASH) and to investigate the mechanism that links mitochondrial dysfunction and hepatic insulin resistance induced by non‐esterified fatty acids (NEFAs). Patients and cows with NASH displayed high blood NEFAs, TNF‐α and IL‐6 concentrations, mitochondrial dysfunction and insulin resistance. The protein levels of peroxisome proliferator‐activated receptor‐γ coactivator‐1α (PGC‐1α), mitofusin‐2 (Mfn‐2) and OXPHOS complexes (human: COI and COIII; cow: COI‐IV) were significantly decreased in patients and cows with NASH. NEFA treatment significantly impaired mitochondrial function and, increased reactive oxygen species (ROS) production, and excessive ROS overactivated the JNK and p38MAPK pathways and induced insulin resistance in cow hepatocytes. PGC‐1α and Mfn‐2 overexpression significantly decreased the NEFA‐induced ROS production and TNF‐α and IL‐6 mRNA expressions, reversed the inhibitory effect of NEFAs on mitochondrial function and attenuated the overactivation of the ROS‐JNK/p38MAPK pathway, alleviated insulin resistance induced by NEFAs in cow hepatocytes and HepG2 cells. These findings indicate that NEFAs induce mitochondrial dysfunction and insulin resistance mediated by the ROS‐JNK/p38MAPK pathway. PGC‐1α or Mfn‐2 overexpression reversed the lipotoxicity of NEFAs on mitochondrial dysfunction and insulin resistance. Our study clarified the mechanism that links hepatic mitochondrial dysfunction and insulin resistance in NASH.

## 1 INTRODUCTION

Non‐alcoholic fatty liver disease (NAFLD), a burgeoning health problem worldwide, is characterized by triglyceride (TG) accumulation in hepatocytes and can progress to NASH, cryptogenic liver cirrhosis and even hepatocellular carcinoma. NAFLD has emerged as one of the most common chronic liver diseases over the past decade, with a prevalence of 20% to 30% in North America and 15% in China.^1^ Interestingly, up to 50% of all dairy cows exhibit hepatic TG accumulation following calving.^2^ More importantly, compelling evidence had indicated that patients with NAFLD and cows with fatty liver both displayed high blood concentrations of NEFAs and hepatic TG accumulation.^3^, ^4^, ^5^, ^6^ A high NEFA level is the pathological basis of fatty liver; however, the detailed molecular mechanisms underlying this pathology are not clearly understood.

The regulation of hepatic lipid metabolism is largely dependent on mitochondria, the primary organelle of cellular adenosine triphosphate (ATP) production, using the energy released from the respiratory chain mediated by OXPHOS complexes (COI, COII, COIII, COIV and COV).^7^ NEFAs are a crucial energy source in the liver and peripheral tissues. However, high level of NEFAs caused lipotoxicity and could impair hepatic mitochondrial function and insulin signalling.^8^, ^9^ Furthermore, studies in humans and experimental models have shown that mitochondrial dysfunction is involved in the development of insulin resistance and NAFLD.^10^, ^11^ A study in rats showed that impairment of the mitochondrial respiratory chain results in mitochondrial ROS overproduction, which in turn causes oxidative damage to mitochondrial structure and function, leading to abnormal changes in intracellular signalling and the development of insulin resistance.^12^, ^13^ The regulation and maintenance of mitochondrial function require regulators, such as peroxisome proliferator‐activated receptor‐γ coactivator‐1α (PGC‐1α) and mitofusin‐2 (Mfn‐2), which play critical roles in regulating mitochondria biogenesis, respiration and OXPHOS.^14^ Besides, studies had shown that adipose PGC‐1α deficiency clearly leaded to systemic deregulation of glucose homoeostasis, and obesity induced by a high‐fat diet (HFD) reduced Mfn‐2 expression in muscle.^15^, ^16^ However, the effects of PGC‐1α and Mfn‐2 on insulin signalling in hepatocytes are still not well understood.

Results from ob/ob mice, mice fed a HFD, patients with NAFLD or type 2 diabetes and L6 skeletal muscle cells have demonstrated that insulin resistance might coexist with mitochondrial alterations such as lower mitochondrial density, OXPHOS gene expression and ATP synthesis.^10^, ^17^, ^18^, ^19^, ^20^ However, these previous studies investigated the mitochondrial dysfunction and insulin resistance in NAFLD and type 2 diabetes mainly using skeletal muscle and rarely using liver tissue, especially in patients with NASH. Recently, a study reported that the adaptation of hepatic mitochondrial function in humans with insulin resistance is lost in NASH,^21^ although the underlying mechanism remains unclear. High level of NEFAs caused lipotoxicity and impaired fatty acid oxidation in mitochondria affects mitochondrial function, contributes to fat accumulation and hepatic steatosis and correlates with impaired insulin signalling. Therefore, it is of great interest to clarify the relevant mechanisms that link high level of NEFAs, mitochondrial dysfunction and hepatic insulin resistance in humans or dairy cows with NASH. Furthermore, the changes in the expression of hepatic mitochondrial OXPHOS complexes in humans and cows with NASH are poorly characterized.

## 2 MATERIALS AND METHODS

### 2.1 Human liver specimens

This study was carried out in accordance with the recommendations of Declaration of Helsinki guidelines and approved by the ethics committee of the First Hospital of Jilin University (20170221). All subjects gave written informed consent in accordance with the Declaration of Helsinki. The protocol was approved by the ethics committee of the First Hospital of Jilin University (2015‐172). The details of the ethic protocol are included in the supporting information. The healthy, normal‐weight research volunteers who participated in these studies were undergoing abdominal surgical procedures such as herniotomy or cholecystectomy. All surgical liver biopsy samples were collected during surgery. Histopathologic diagnoses for NASH were undertaken in accordance with characteristic histological features.^22^ Briefly, patients with NAFLD displayed isolated hepatic steatosis and patients with NASH displayed hepatic steatosis, inflammation and hepatocellular ballooning. No patient tested positive for hepatitis B virus, hepatitis C virus or human immunodeficiency virus infections. Patients with other causes of chronic liver disease and renal dysfunction or those receiving potentially hepatotoxic drugs were excluded. In addition, the patients consumed less than 20 g of alcohol per day. The clinical characteristics are shown in Table S1.

### 2.2 Animals

All animal experiments were carried out in accordance with the recommendations of the Administration of Affairs Concerning Experimental Animals in China. The protocol was approved by the Animal Welfare and Research Ethics Committee at Jilin University (2016 clinical trial [2016‐101]). Twenty healthy Holstein cows and ten cows with fatty liver (in the second to fourth lactation) were selected from a 2000‐cow dairy farm located in Changchun City, China. Cows with NASH were selected based on histopathologic diagnoses. Blood samples were extracted from the jugular vein to obtain serum. Hepatic samples were collected by an experienced veterinarian using a liver puncture needle between the cows’ 10th and 11th ribs. The clinical characteristics are shown in Table S2. During the experimental work, the cows were housed in a climate‐controlled barn in individual tie stalls to reduce environmental effects.

### 2.3 Hepatocyte culture and treatment

All animal experiments were carried out in accordance with the recommendations of the Administration of Affairs Concerning Experimental Animals in China. The protocol was approved by the Animal Welfare and Research Ethics Committee at Jilin University (2016 clinical trial [2016‐101]). Cow hepatocytes were isolated from newborn male Holstein calves using a two‐step perfusion method published previously.^23^, ^24^ The cell density was adjusted to 1.5 × 10^6^ cells/mL using adherent medium (RPMI‐1640 basic medium supplemented with 10% newborn calf serum, 10^−6^ mol/L insulin, 10^−6^ mol/L dexamethasone, 10 μg/mL vitamin C) and incubated at 37°C in 5% CO_2_. After 5 hours, the adherent medium was replaced with growth medium containing 10% newborn calf serum. The growth medium was replaced every 24 hours. Human hepatocarcinoma HepG2 cells (ATCC, Rockville, MD, USA) were maintained in low‐glucose DMEM supplemented with 10% foetal bovine serum (FBS), 100 U/mL penicillin and 100 mg/mL streptomycin in a humidified atmosphere containing 5% CO_2_ at 37°C. A total 1.0 × 10^6^ cells were seeded into a 6‐well plate.

The NEFA treatment concentration and components (oleic acid, linoleic acid, palmitic acid, stearic acid and palmitoleic acid) used in this study were according to the blood concentrations and components of fatty acids in the cows and patients with NASH. The stock NEFA (52.7 mmol/L) solution included oleic acid (22.9 mmol/L), linoleic acid (2.6 mmol/L), palmitic acid (16.8 mmol/L), stearic acid (7.6 mmol/L) and palmitoleic acid (2.8 mmol/L). Cow hepatocytes were maintained in RPMI‐1640 basic medium and treated with 0, 0.6, 1.2 or 2.4 mmol/L NEFAs for 9 hours. HepG2 cells were maintained in low‐glucose DMEM medium and treated with 0.8 mmol/L NEFAs for 9 hours. To overexpress PGC‐1α or Mfn‐2 in vitro, cow hepatocytes and HepG2 cells were infected with adenoviruses overexpressing PGC‐1α (Ad‐PGC‐1α) or Mfn‐2 (Ad‐Mfn‐2) (Hanbio, Shanghai, China) for 48 hours according to the manufacturer's instructions. To evaluate the insulin signalling pathway, cells were treated with 100 nmol/L insulin for 30 minutes. The cell treatment and detailed grouping were shown in the figure legends.

### 2.4 Serum malondialdehyde and superoxide dismutase measurements

The content of malondialdehyde (MDA) and activity of superoxide dismutase (SOD) were measured using biochemical kits (Nanjing Jiancheng Institute of Bioengineering, Nanjing, China). The MDA measurement was based on the reaction with thiobarbituric acid in acidic medium at 95°C at 533 nm. The MDA concentration was calculated from the standard curve and expressed as nmol/mL serum. SOD activity was determined spectrophotometrically at 550 nm by use of xanthine and xanthine oxidase systems. One unit of SOD activity was defined as the amount of enzyme required to cause 50% inhibition of the xanthine and xanthine oxidase system reaction of 1 mg haemoglobin. SOD activity was expressed as U/mg of haemoglobin.

### 2.5 Western blotting assay

Total protein from liver tissues, cow hepatocytes and HepG2 cells was extracted using a protein extraction kit (Sangon Biotech Co., Ltd., Shanghai, China) according to the supplier's protocol. A total 20 μg of protein from each sample were separated by polyacrylamide gel electrophoresis and electrotransferred onto PVDF membranes. Then, the membranes were blocked and hybridized with antibodies against phosphorylated‐protein kinase B (p‐AKT; Cat: 4060), AKT (Cat: 9272), phosphorylated‐glycogen synthesis kinase 3 beta (p‐GSK3β; Cat: 9336), c‐Jun N‐terminal kinase (JNK; Cat: 9252), p‐JNK (Cat: 9251), p38 mitogen‐activated protein kinase (p38MAPK; Cat: 8690), p‐p38MAPK (Cat: 4370; Cell Signaling Technology, Danvers, MA, USA), GSK3β (Cat: ab69739), PGC‐1α (Cat: ab54481), Mfn‐2 (Cat: ab56889), COI (Cat: 14713), COII (Cat: 14715), COIII (Cat: ab14745), COIV (Cat: ab14744), COV (Cat: ab14730), nuclear respiratory factor 1 (NRF1; Cat: ab154269), insulin receptor substrate 2 (IRS2; Cat: ab3690), p‐IRS2 (Cat: ab134101), β‐actin (Cat: ab8226; Abcam, Cambridge, MA, USA) and mitochondrial transcription factor A (TFAM; Cat: sc166965; Santa Cruz Biotechnology, Santa Cruz, CA) and then incubated with a secondary antibody. Immunoreactive bands were analysed using an enhanced chemiluminescence solution (Pierce Biotechnology Inc., Chicago, USA). Finally, the bands were imaged using a Protein Simple imager (ProteinSimple, Santa Clara, CA, USA).

### 2.6 RNA extraction and real‐time RT‐PCR

Total RNA from liver tissue and hepatocytes was extracted with TRIzol reagent (TaKaRa Biotechnology Co., Ltd., Tokyo, Japan) according to the supplier's protocol. A total of 3 μg of RNA was transcribed into cDNA using PrimeScript Reverse Transcriptase (TaKaRa Biotechnology Co., Ltd.). mRNA expression levels were evaluated by qRT‐PCR analysis using FS Universal SYBR Green Real Master (Roche Diagnostics Ltd, Lewes, UK) on a 7500 Real‐time PCR System (Applied Biosystems, Inc., Foster City, CA). The relative expression of each target gene was normalized to that of β‐actin. The primers for each gene were designed using Primer Express software (Applied Biosystems) and are shown in Table S3.

### 2.7 Determination of ATP concentration, membrane potential and ROS concentration

The ATP concentration of hepatocytes was measured using an ATP assay kit (Beyotime Institute of Biotechnology, Shanghai, China) following the manufacturer's protocol. Briefly, the cells were lysed in lysis buffer and centrifuged at 4°C and 12 000 *g* for 10 minutes. The supernatants were used for ATP detection using a Centro LB960 luminometer (Berthold Technologies, Bad Wildbad, Germany). The concentration of ATP was calculated in accordance with a standard curve and converted into nmol/mg protein.

For the quantitative estimation of mitochondrial membrane potential, hepatocytes were incubated with 10 μmol/L rhodamine 123 (Beyotime Institute of Biotechnology) for 30 minutes in the dark, and the fluorescence intensity of the dye was determined by flow cytometry (Becton‐Dickinson, Mountain View, USA).

The intracellular ROS concentrations were measured using the peroxide‐sensitive fluorescent probe 2′7′‐dichlorofluorescein diacetate (DCFH‐DA) (Beyotime Biotechnology Inc., Nantong, China). The cells were exposed to serum‐free medium containing 10 μmol/L DCFH‐DA and propidium iodide in the dark for 30 minutes and then washed three times with cold PBS. The fluorescence was measured by flow cytometry (Becton‐Dickinson, Mountain View, USA).

### 2.8 Histological analysis

Liver tissue haematoxylin and eosin (HE) staining was conducted after the tissue was fixed in methanol‐free 4% paraformaldehyde for 24‐36 hours. For HE staining, the paraffin‐embedded specimens were sectioned at 5 μm and then stained. The ultrastructural characteristics of mitochondria in treated and control cells were visualized by transmission electron microscopy. Liver tissue and cow hepatocytes were fixed, dehydrated and infiltrated. Ultrathin sections (50 nm) were cut and stained with 4% uranyl acetate and 0.2% lead citrate. Finally, the sections were examined with an H‐7650 electron microscope.

### 2.9 Detection of blood biochemical indicators

Glucose, NEFAs, insulin, TG, aspartate transaminase (AST), alanine aminotransferase (ALT), gamma‐glutamyl transpeptidase (γ‐GT), tumour necrosis factor α (TNF‐α) and interleukin‐6 (IL‐6) were measured using biochemical or ELISA kits (Beijing Strong Biotechnologies, Inc., Beijing, China; R&D Systems, Minneapolis, USA) according to the supplier's protocol. Haemoglobin A1_c_ (HbA1_c_) levels were determined using high‐performance liquid chromatography. The homoeostasis model assessment of insulin resistance (HOMA‐IR) was calculated according to the following formula: (fasting glucose levels [mmol/L]) × (fasting serum insulin [mU/L])/22.5. Body mass index (BMI) was calculated according to the following formula: (weight [kg])/(height [m] × height [m]).

### 2.10 Measurement of liver TG contents

Approximately 30 mg of liver tissue was chopped and homogenized in 0.5 mL of 5% Triton‐X100 in water. The homogenized tissue was heated in a water bath (85°C) for 3 minutes. After cooling at room temperature, the samples were vortexed and centrifuged at 2000 *g* for 5 minutes at 4°C. The supernatant was collected and stored at −80°C before the TG assay. The TG contents were measured using an enzymatic kit (Applygen Technologies Inc., Beijing, China) following the manufacturer's instructions.

### 2.11 Statistical analysis

The results are expressed as the means ± standard deviation (SD). SPSS software (SPSS Incorporated, Chicago, IL, USA) version 19.0 was used to analyse the data. Statistical significance was calculated using Student's *t* test in cases of comparisons between two groups and one‐way ANOVA in cases of comparisons among more than two groups. A *P* value less than .05 was considered statistically significant, and values less than 0.01 were considered highly significant.

## 3 RESULTS

### 3.1 The evaluation of hepatic insulin signalling in patients and cows with NASH

**Figure 1:**
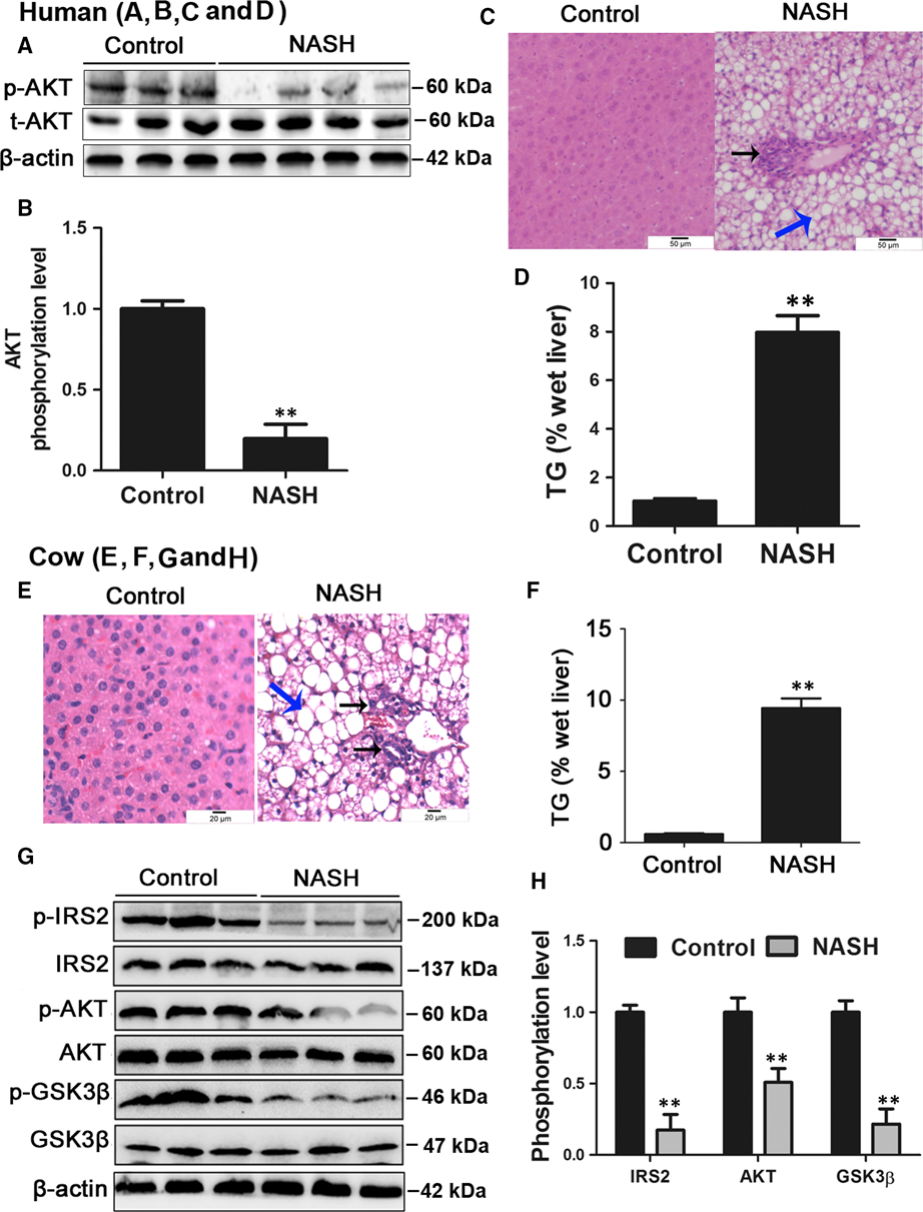
Patients and cows with NASH displayed insulin resistance and hepatic steatosis. A, B, The hepatic phosphorylation level of AKT (p‐AKT/AKT) in patients with NASH and controls. C, HE staining of liver histology from control and NASH patient (original magnification ×20). Black arrow, inflammatory infiltration; Blue arrow, lipid droplet. D, Hepatic TG content in patients with NASH and controls. E, HE staining of liver histology from control and NASH cows (original magnification ×40). Black arrow, inflammatory infiltration; Blue arrow, lipid droplet. F, Hepatic TG content in control and NASH cows. G, H, The hepatic phosphorylation levels of AKT, GSK3β and IRS2 in control and NASH cows. All data are expressed as mean ± SD. *P < .05 and **P < .01 compared with control group. Subjects: eight patients with NASH and six healthy subjects. Cows: twenty healthy Holstein cows and ten cows with NASH. Each treatment was repeated 8 times. NASH, non‐alcoholic steatohepatitis; AKT, protein kinase B; TG, triglyceride; GSK3β, glycogen synthesis kinase 3 beta; IRS2, insulin receptor substrate 2; SD, standard deviation

The baseline characteristics of control and NASH subjects are shown in Table S1. The blood levels of fasting free fatty acids, glucose, TGs, insulin and HbA1_c_ and the HOMA‐IR and BMI of patients with NASH were significantly higher than those of controls. The hepatic phosphorylation level of AKT (p‐AKT/AKT, at Ser473) was significantly lower in patients with NASH than in controls (Figure 1A and B). Furthermore, the blood concentration of IL‐6, TNF‐α, ALT, AST and γ‐GT was also significantly increased in patients with NASH (Table S1). Besides, the ratio of AST/ALT was significantly increased in the serum of patients with NASH (Table S1). Moreover, HE staining showed that patients with NASH exhibited significant lipid accumulation and inflammatory infiltration (Figure 1C). The hepatic TG content in patients with NASH was also significantly higher than that of controls (Figure 1D). These results indicate that patients with NASH have clear systemic and liver insulin resistance, inflammation, lipid accumulation and hepatic injury.

Similar to NASH patients, HE staining of liver tissue from cows with NASH showed significant lipid accumulation and inflammatory infiltration (Figure 1E). Dairy cows with NASH also displayed high blood concentrations of NEFAs, TNF‐α, IL‐6, ALT, AST and γ‐GT (Table S2) and hepatic TG accumulation (Figure 1F). Moreover, the phosphorylation levels of IRS2 (p‐IRS2/IRS2, at Ser731), AKT (at Ser473) and GSK3β (p‐GSK3β/GSK3β, at Ser9) were significantly decreased in cows with NASH (Figure 1G and H). These results indicate that cows with NASH also exhibit systemic and liver insulin resistance, inflammation, lipid accumulation and hepatic injury.

### 3.2 Patients and cows with NASH display mitochondrial dysfunction

**Figure 2:**
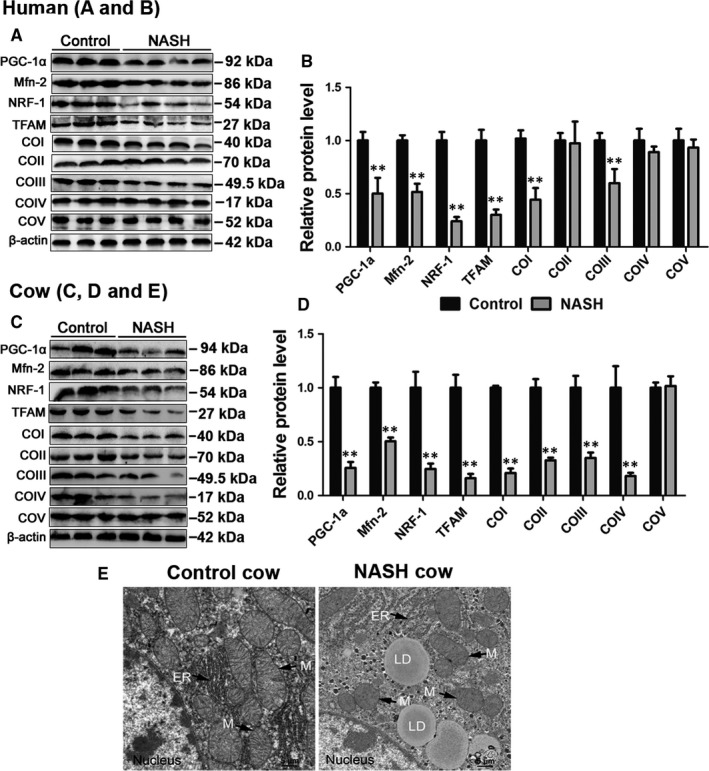
Patients and cows with NASH displayed mitochondrial dysfunction. A, B, Western blot analysis and quantification of hepatic PGC‐1α, Mfn‐2, NRF‐1, TFAM and five representative subunits of OXPHOS complexes in NASH patients and controls. C, D, Western blot analysis and quantification of hepatic PGC‐1α, Mfn‐2, NRF1, TFAM in NASH cows and controls. E, Representative TEM images of liver sections derived from healthy cows and NASH cows at ×3000 magnification. Scale bars, 5 μm. M, mitochondria; N, nucleus; LD, lipid droplet. All data are expressed as mean ± SD. *P < .05 and **P < .01 compared with normal liver group. Subjects: eight patients with NASH and six healthy subjects. Animals: twenty healthy Holstein cows and ten cows with NASH. Each treatment was repeated 8 times. PGC‐1α, peroxisome proliferator‐activated receptor‐γ coactivator‐1α; Mfn‐2, mitofusin‐2; NRF1, nuclear respiratory factor 1; TFAM, mitochondrial transcription factor A; TEM, transmission electron microscope

The mitochondrial function regulators PGC‐1α, Mfn‐2, NRF1 and TFAM play critical roles in regulating mitochondria biogenesis, respiration and OXPHOS.^25^, ^26^, ^27^ Our in vivo data showed that the protein levels of PGC‐1α, Mfn‐2, NRF‐1 and TFAM were significantly decreased in patients and cows with NASH (Figure 2A‐D). Furthermore, we found that the protein levels of COI‐V were lower in patients with NASH than in controls, and COI and COIII were significantly decreased (Figure 2A,B). These results indicate that patients with NASH display defects in the hepatic mitochondrial respiratory chain. In addition, the protein expression of COI‐IV was significantly lower in cows with NASH than in the control cows (Figure 2C,D). Ultrastructural analysis revealed a uniform accumulation of cytosolic lipid droplets in the hepatocytes of cows with NASH, indicative of microvesicular steatosis. In addition, the mitochondria displayed swollen and crest ruptures (Figure 2E). These results further indicate that patients or cows with NASH display severe mitochondrial dysfunction.

### 3.3 High NEFAs cause mitochondrial dysfunction and insulin resistance in cow hepatocytes in vitro

Excessive NEFA flux into the liver is the pathological basis of fatty liver, inducing a series of pathological changes, such as oxidative stress, inflammation, hepatic steatosis and insulin resistance.^3^, ^28^, ^29^ We therefore attempted to investigate the relevant mechanisms that link NEFAs, mitochondrial dysfunction and hepatic insulin resistance in vitro via cow hepatocytes.

**Figure 3:**
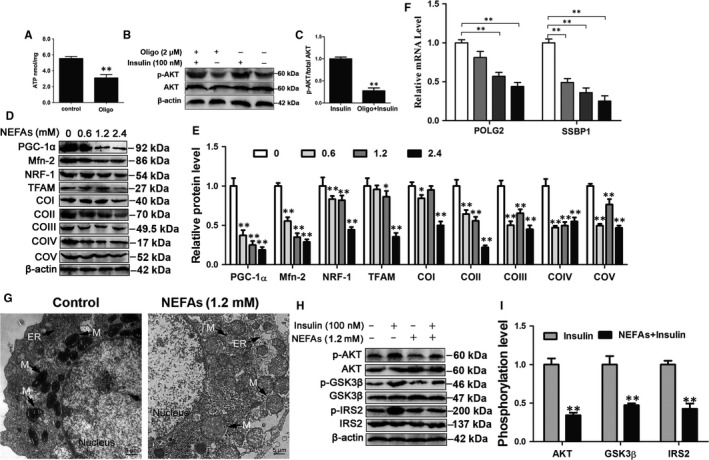
NEFA treatment induced mitochondrial dysfunction and insulin resistance. A, The ATP content. Cow hepatocytes were treated with 2 μmol/L oligo for 6 h. B and C, Western blot analysis and quantification of p‐AKT and AKT in cow hepatocytes. Cow hepatocytes were treated with 2 μmol/L Oligo for 6 h and then treated with or without 100 nmol/L insulin for 30 min. D, E and F, Hepatocytes were treated with 0, 0.6, 1.2 and 2.4 mmol/L NEFAs, respectively. D and E, Western blot analysis and quantification of PGC‐1α, Mfn‐2, NRF1, TFAM and five representative subunits of OXPHOS complexes. F, The mRNA levels of POLG2 and SSBP1. G, Representative TEM images of hepatocytes treated with 1.2 mmol/L NEFAs at ×2500 magnification. Scale bar, 5 μm. H and I, Cow hepatocytes were treated with 1.2 mmol/L NEFAs in the absence or presence of 100 nmol/L insulin. The phosphorylation of AKT, GSK3β and IRS2 was analysed by Western blots. All data represent the mean ± SD. *P < .05 and **P < .01 compared with no treatment group. Each treatment was repeated 8 times. NEFAs, non‐esterified fatty acids; OXPHOS, oxidative phosphorylation; POLG2, gamma DNA polymerase; SSBP1, single‐strand DNA binding protein 1

To investigate the relationship between insulin resistance and mitochondrial dysfunction, cow hepatocytes were treated with oligomycin, a specific inducer of mitochondrial dysfunction. Oligomycin treatment significantly decreased the ATP content and the phosphorylation level of AKT (Figure 3A‐C). Accordingly, these results indicate that mitochondrial dysfunction is associated with insulin resistance in cow hepatocytes.

To investigate the effect of NEFAs on mitochondrial function, cow hepatocytes were treated with different concentrations of NEFAs. The protein expression of PGC‐1α, Mfn‐2, NRF‐1, TFAM and COI‐V showed a downward trend and was significantly lower in 1.2 and 2.4 mmol/L NEFA‐treated cow hepatocytes than in control cells (Figure 3D and E). To further evaluate mitochondrial function, we detected the mRNA expression of gamma DNA polymerase (POLG2, the accessory subunit POLG2) and single‐strand DNA binding protein 1 (SSBP1), which respond to mtDNA replication and repair.^30^ The mRNA level of POLG2 was significantly decreased in the 1.2 and 2.4 mmol/L NEFA‐treated groups, and SSBP1 was significantly decreased in the 0.6, 1.2 and 2.4 mmol/L NEFA‐treated groups (Figure 3F). Mitochondria in NEFA‐treated hepatocytes displayed an increased number of disarrayed cristae and a reduced matrix electron density (Figure 3G). Taken together, these results indicate that treatment with high level of NEFA treatment induces mitochondrial dysfunction in cow hepatocytes. Furthermore, the phosphorylation levels of AKT, GSK3β and IRS2 were significantly decreased in NEFA‐treated cells (Figure 3H and I). These results suggest that treatment with high level of NEFAs markedly impairs the insulin signalling pathway.

### 3.4 NEFA‐induced mitochondrial dysfunction impairs the insulin signalling pathway through the ROS‐JNK/p38MAPK pathway

**Figure 4:**
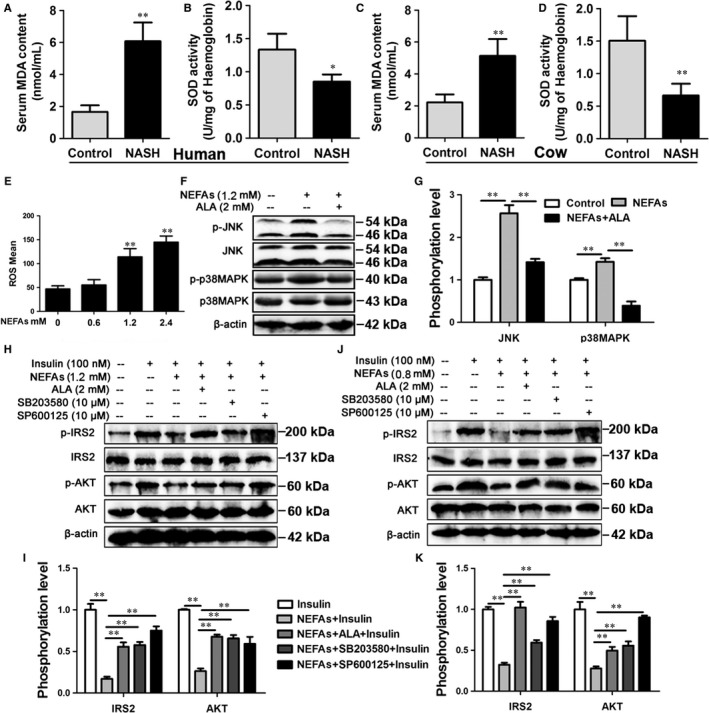
NEFA‐induced insulin resistance through ROS‐JNK/p38MAPK pathway. A and B, Subjects: eight patients with NASH and six healthy subjects. A, The blood level of MDA in NASH and control subjects. B, The blood activity of SOD in NASH and control subjects. C and D, Cows: twenty healthy Holstein cows and ten cows with NASH. C, The blood level of MDA in NASH and control cows. D, The blood activity of SOD in NASH and control cows. E, The ROS content in the NEFA‐treated cow hepatocytes. The cow hepatocytes were treated with 0, 0.6, 1.2 and 2.4 mmol/L NEFAs, respectively. F and G, Western blot analysis and quantification of JNK, p‐JNK, p38MAPK and p‐p38MAPK. The cow hepatocytes were treated with 1.2 mmol/L NEFAs in the absence or presence of 2 mmol/L ALA. H and I, Western blot analysis and quantification of IRS2, p‐IRS2, AKT and p‐AKT. Cow hepatocytes were treated with 1.2 mmol/L NEFAs, 100 nmol/L insulin, 2 mmol/L ALA, 10 μmol/L JNK inhibitor SP600125 and 10 μmol/L p38MAPK inhibitor SB203580, respectively. G and K, Western blot analysis and quantification of IRS2, p‐IRS2, AKT and p‐AKT. HepG2 cells were treated as described in the (H). All data represent the mean ± SD. *P < .05 and **P < .01 compared with no treatment group. Each treatment was repeated 8 times. MDA, malondialdehyde; SOD, superoxide dismutase; JNK, c‐Jun N‐terminal kinase; p38MAPK, p38 mitogen‐activated protein kinase; ALA, alpha‐lipoic acid; HepG2, hepatocellular carcinoma

Oxidative stress is a significant feature of patients and cows with NASH.^3^, ^31^ To evaluate oxidative stress in our experiments, the blood MDA level and SOD activity were detected. The blood MDA level was markedly higher in patients and cows with NASH than in controls (Figure 4A and C), but SOD activity was significantly lower (Figure 4B and D), indicating that patients and cows with NASH display severe oxidative stress. Our data demonstrated that NEFA treatment could induce mitochondrial dysfunction. As shown in Figure 4E, the ROS content was significantly increased in 1.2 and 2.4 mmol/L NEFA‐treated cow hepatocytes (Figures 4E and S1). These data demonstrate that patients and cows with NASH display oxidative stress and that a high level of NEFAs can cause oxidative stress in hepatocytes.

Reactive oxygen species‐induced insulin resistance is mediated by various signalling pathways, including the JNK and p38 MAPK pathways.^20^, ^32^ Our results showed that NEFA treatment markedly increased the phosphorylation levels of JNK (p‐JNK/JNK, at Thr183 and Tyr185) and p38MAPK (p‐p38MAPK/p38MAPK, at Thr180 and Tyr182) and decreased the phosphorylation levels of IRS2 and Akt in cow hepatocytes (Figure 4F‐I). Alpha‐lipoic acid (ALA) is an antioxidant that acts on mitochondria.^33^ We found that ALA treatment could decrease the phosphorylation levels of JNK and p38MAPK and improve insulin resistance (Figure 4F‐I). These results indicate that NEFAs can induce the excessive mitochondrial ROS production and then overinduce the JNK and p38MAPK pathways, thus impairing the insulin pathway. To further investigate the role of JNK and p38MAPK in ROS‐induced insulin resistance, cow hepatocytes were treated with the JNK inhibitor SP600125 and the p38MAPK inhibitor SB203580. As expected, inhibition of JNK or p38MAPK markedly improved the insulin resistance induced by NEFAs in cow hepatocytes (Figure 4H and I). Furthermore, the ROS‐JNK/p38MAPK pathway was also found to mediate NEFA‐induced insulin resistance in HepG2 cells (Figure 4G and K). Taken together, these findings indicate that NEFA treatment results in excessive mitochondrial ROS production and induces the overactivation of the JNK and p38MAPK pathways, thereby inducing insulin resistance in hepatocytes.

### 3.5 PGC‐1α overexpression improves mitochondrial dysfunction and insulin resistance induced by NEFAs in cow hepatocytes

**Figure 5:**
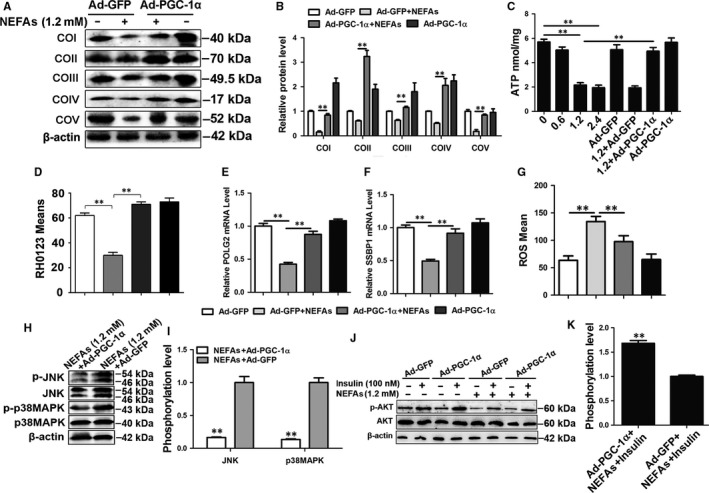
PGC‐1α overexpression improved mitochondrial dysfunction and insulin resistance induced by NEFAs in cow hepatocytes. A, B, D, E, F and G, Cells were infected with Ad‐GFP or Ad‐PGC‐1α and then treated with/without 1.2 mmol/L NEFAs, respectively. A and B, Western blot analysis and quantification of five representative subunits of OXPHOS complexes. C, ATP content. Four groups were treated with different concentration of NEFAs (0, 0.6, 1.2, 2.4 mmol/L), other four groups were infected with Ad‐GFP or Ad‐PGC‐1α and then treated with/without 1.2 mmol/L NEFAs, respectively. D, Membrane potential. E and F, The mRNA levels of POLG2 and SSBP1. G, ROS content. H and I, Western blot analysis and quantification of JNK, p‐JNK, p38MAPK and p‐p38MAPK. Cow hepatocytes were infected with Ad‐PGC‐1α or Ad‐GFP and then treated with 1.2 mmol/L NEFAs. J and K, Western blot analysis and quantification of AKT and p‐AKT. Cow hepatocytes were infected with Ad‐PGC‐1α or Ad‐GFP and then treated with/without 1.2 mmol/L NEFAs and/or 100 nmol/L insulin, respectively. All data represent the mean ± SD. *P < .05 and **P < .01 compared with no treatment group. Each treatment was repeated 8 times. Ad‐GFP, green fluorescent protein adenoviral vectors; Ad‐PGC‐1α, PGC‐1α adenoviral vectors; ROS, reactive oxygen species

PGC‐1α plays a critical role in regulating mitochondria function.^34^ To determine the role of PGC‐1α in the mitochondrial dysfunction and insulin resistance induced by NEFAs, PGC‐1α was overexpressed by adenovirus (Figure S2). As shown in Figure 5A, PGC‐1α overexpression significantly reversed the inhibitory effect of NEFAs on the protein expression of COI, COII, COIII, COIV and COV (Figure 5A,B), increased ATP production (Figure 5C) and increased membrane potential (Figures 5D and S3A). The mRNA expression of SSBP1 and POLG2 also significantly increased in the PGC‐1α overexpression group (Figure 5E and F). These results indicate that PGC‐1α overexpression significantly ameliorates the mitochondrial dysfunction induced by NEFAs in cow hepatocytes.

Importantly, PGC‐1α overexpression significantly decreased NEFA‐induced excessive mitochondrial ROS production (Figures 5G and S3B). Subsequently, to further evaluate the improvement effect of PGC‐1α overexpression on insulin resistance, the phosphorylation levels of JNK, p38MAPK and AKT were detected by Western blotting. The JNK and p38MAPK phosphorylation levels were significantly lower in the Ad‐PGC‐1α + NEFA group than in the NEFA group (Figure 5H and I), but the AKT phosphorylation level was significantly higher in the former group (Figure 5J and K). Altogether, these results indicate that PGC‐1α overexpression can attenuate the activation of the ROS‐JNK/p38MAPK pathway induced by NEFAs and improve insulin resistance in cow hepatocytes.

### 3.6 Mfn‐2 overexpression ameliorates mitochondrial dysfunction and insulin resistance induced by NEFAs in cow hepatocytes

**Figure 6:**
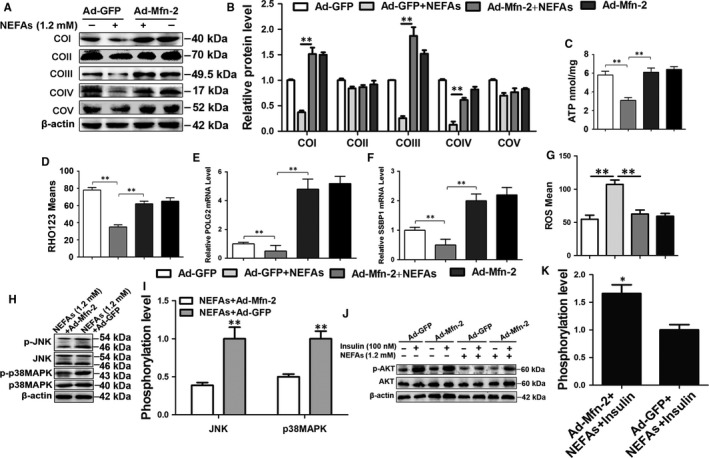
Mfn‐2 overexpression improved mitochondrial dysfunction and insulin resistance induced by NEFAs in cow hepatocytes. A‐G, Cells were infected with Ad‐GFP or Ad‐ Mfn‐2 and then treated with/without 1.2 mmol/L NEFAs, respectively. A and B, Western blot analysis and quantification of five representative subunits of OXPHOS complexes. C, ATP content. D, Membrane potential. E and F, The mRNA levels of POLG2 and SSBP1. G, ROS content. H and I, Western blot analysis and quantification of JNK, p‐JNK, p38MAPK and p‐p38MAPK. Cow hepatocytes were infected with Ad‐Mfn‐2 or Ad‐GFP and then treated with 1.2 mmol/L NEFAs. J and K, Western blot analysis and quantification of AKT and p‐AKT. Cow hepatocytes were infected with Ad‐Mfn‐2 or Ad‐GFP and then treated with/without 1.2 mmol/L NEFAs and/or 100 nmol/L insulin, respectively. All data represent the mean ± SD. *P < .05 and **P < .01 compared with no treatment group. Each treatment was repeated 8 times

To determine the roles of Mfn‐2 in the improvement of mitochondrial dysfunction and insulin resistance induced by NEFAs, Mfn‐2 was overexpressed by adenovirus (Figure S4). Mfn‐2 overexpression in cow hepatocytes significantly increased the protein expression of COI, COIII and COIV (Figure 6A and B), ATP content and mitochondrial membrane potential (Figures 6C,D and S5A). The mRNA expression of SSBP1 and POLG2 also significantly increased in the Mfn‐2 overexpression group (Figure 6E and F). These results indicate that Mfn‐2 overexpression significantly improves mitochondrial dysfunction induced by NEFAs.

Similar to PGC‐1α overexpression, Mfn‐2 overexpression significantly decreased the ROS content (Figures 6G and S5B) and JNK and p38MAPK phosphorylation levels (Figure 6H and I) and increased the AKT phosphorylation level in the Ad‐Mfn‐2 + NEFA group compared with the NEFA group (Figure 6J and K). Their results indicate that Mfn‐2 overexpression can also improve insulin resistance by attenuating the overactivation of the ROS‐JNK/p38MAPK pathway induced by NEFAs in cow hepatocytes.

### 3.7 PGC‐1α or Mfn‐2 overexpression improves mitochondrial dysfunction and insulin resistance in HepG2 cells

**Figure 7:**
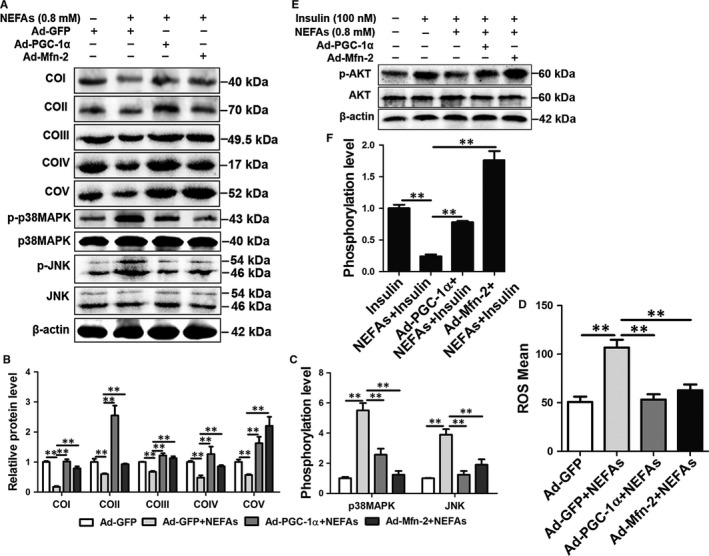
Overexpression of PGC‐1α or Mfn‐2 improved mitochondrial dysfunction and insulin resistance induced by NEFAs in HepG2 cells. A‐D, HepG2 cells were infected with Ad‐GFP, Ad‐PGC‐1α and Ad‐Mfn‐2, and then treated with 0.8 mmol/L NEFAs. A, B and C, Western blot analysis and quantification of JNK, p‐JNK, p38MAPK, p‐p38MAPK and five representative subunits of OXPHOS complexes. D, ROS content. E and F, Western blot analysis and quantification of p‐AKT and AKT. HepG2 cells were infected with Ad‐GFP, Ad‐PGC‐1α, and Ad‐Mfn‐2, and then treated with 0.8 mmol/L NEFAs and/or 100 nmol/L insulin. All data represent the mean ± SD. *P < .05 and **P < .01 compared with no treatment group. Each treatment was repeated 8 times

To further investigate the beneficial role of PGC‐1α and Mfn‐2 on mitochondrial dysfunction and insulin resistance, HepG2 cells were infected with adenoviruses overexpressing PGC‐1α or Mfn‐2 and then treated with NEFAs. PGC‐1α or Mfn‐2 overexpression significantly increased the protein expression of COI‐COV (Figure 7A and B) and decreased the NEFA‐induced ROS content (Figures 7D and S6). Furthermore, the JNK and p38MAPK phosphorylation levels were significantly decreased in the Ad‐PGC‐1α + NEFA group and Ad‐Mfn‐2 + NEFA group compared with the NEFA group (Figure 7A and C), and the AKT phosphorylation level was markedly increased (Figure 7E and F). These results further suggest that overexpression of PGC‐1α or Mfn‐2 improves mitochondrial dysfunction and insulin resistance induced by NEFA treatment in HepG2 cells.

### 3.8 PGC‐1α or Mfn‐2 overexpression alleviates lipid accumulation and decreases inflammatory factors expression in cow hepatocytes and HepG2 cells

**Figure 8:**
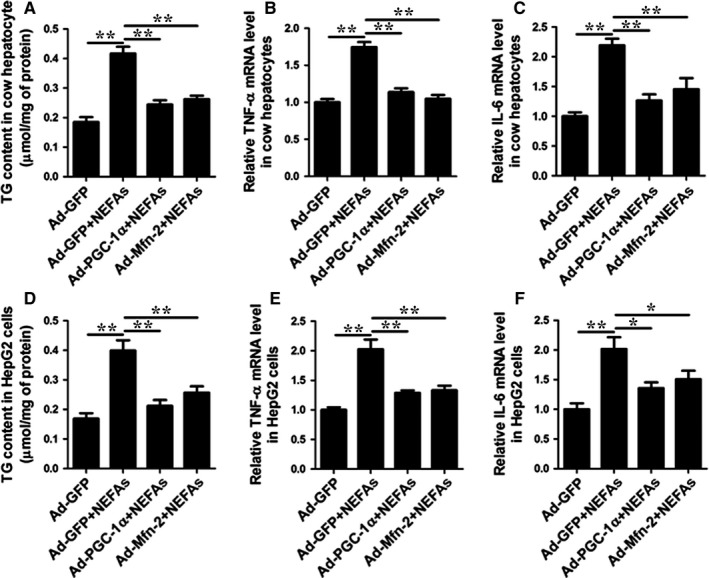
PGC‐1α or Mfn‐2 overexpression alleviates lipid accumulation and decreases inflammatory factors expression in cow hepatocytes and HepG2 cells. A‐C, Cow hepatocytes were infected with Ad‐GFP, Ad‐PGC‐1α and Ad‐Mfn‐2, and then treated with 1.2 mmol/L NEFAs. A, TG content in cow hepatocytes. B, mRNA levels of TNF‐α in cow hepatocytes. C, mRNA levels of IL‐6 in cow hepatocytes. D‐F, HepG2 cells were infected with Ad‐GFP, Ad‐PGC‐1α and Ad‐Mfn‐2, and then treated with 0.8 mmol/L NEFAs. D, TG content in HepG2 cells. E, mRNA levels of TNF‐α in HepG2 cells. F, mRNA levels of IL‐6 in HepG2 cells. All data represent the mean ± SD. *P < .05 and **P < .01 compared with no treatment group. Each treatment was repeated 8 times

To further investigate the beneficial effect of PGC‐1α and Mfn‐2 on lipid accumulation and inflammatory factors expression, cow hepatocytes and HepG2 cells were infected with adenoviruses overexpressing PGC‐1α or Mfn‐2 and then treated with NEFAs. The TG content was significantly increased in cow hepatocytes and HepG2 cells treated with NEFAs (Figure 8A and D). However, PGC‐1α or Mfn‐2 overexpression markedly down‐regulated the NEFA‐induced TG content in cow hepatocytes and HepG2 cells (Figure 8A and D). Furthermore, we investigated the expression of the inflammatory factors TNF‐α and IL‐6 in cow hepatocytes and HepG2 cells treated with NEFAs. The mRNA levels of TNF‐α and IL‐6 were significantly higher in NEFA‐treated cells than in control cells (Figure 8B,C,E and F). Besides, the mRNA levels of TNF‐α and IL‐6 were significantly decreased in the Ad‐PGC‐1α + NEFA group and Ad‐Mfn‐2 + NEFA group compared with the NEFA group in cow hepatocytes and HepG2 cells (Figure 8B,C,E and F). Taken together, these results suggest that PGC‐1α or Mfn‐2 overexpression alleviates lipid accumulation and decreases inflammatory factors expression in cow hepatocytes and HepG2 cells.

## 4 DISCUSSION

Fatty liver is a major metabolic disorder in humans and perinatal dairy cows. Studies have focused on the molecular pathogenesis of NASH in humans mainly using rodent models. Compared with rodent models, dairy cows with NASH have advantages in studies of the molecular pathogenesis of NASH. First, cows are one of the few animals that exhibit naturally occurring fatty liver. Second, patients and cows with NASH both display high NEFA levels, hepatic steatosis, hepatic insulin resistance, oxidative stress and inflammation. In a study by Straub et al, cow and human liver samples were used to investigate the mechanism of hepatic steatosis.^35^ We therefore chose cows with NASH and cow hepatocytes as the animal and cell models to investigate the mechanism of mitochondrial dysfunction and insulin resistance.

Mitochondrial dysfunction is a potential cause of hepatic insulin resistance and steatosis. Until now, few studies have evaluated mitochondrial dysfunction in the livers of human subjects. Pirez‐Cameras et al demonstrated that activities of OXPHOS complexes (COI‐V) were markedly decreased in the livers of patients with NASH.^36^ Koliaki et al found that the protein levels of COI, III, IV and V were lower in patients with NASH than in controls after correcting for mitochondrial content.^21^ In our study, we found that the hepatic protein levels of COI‐V showed a decreasing trend, and COI and III were significantly lower in patients with NASH than in controls. Furthermore, the hepatic protein levels of COI‐IV were significantly decreased in cows with NASH. These results indicate that patients and cows with NASH display impaired mitochondrial OXPHOS complexes. In addition, the above studies and our data suggest that there are discrepancies in the expression of hepatic OXPHOS complexes in NASH in humans, as well as among different species. In addition, result showed that the ratio of AST/ALT was significantly increased in the serum of patients with NASH. This result was consistent with previous studies.^37^, ^38^ However, Guo et al reported that the AST/ALT ratio was significantly increased in mice fed with 4% alcohol.^39^ The discrepancies in the hepatic protein levels of COI‐IV and the AST/ALT ratio between previous studies and our study could be due to differences in samples, the degree of hepatic pathological damage, nutritional factors or genetic factors.

Impaired mitochondrial OXPHOS complexes further decreased the mitochondrial fatty acid β‐oxidation and enhanced TG accumulation. Subsequently, hepatic fatty acid and TG overload can directly inhibit several enzymes involved in the mitochondrial respiratory chain.^26^ Our results showed that NEFA treatment resulted in markedly decreased OXPHOS complexes, POLG2 and SSBP1 expression and ATP content and impaired mitochondria structure in hepatocytes. Furthermore, we also found that insulin resistance coexisted with mitochondrial dysfunction in patients and cows with NASH, and NEFA treatment impaired the insulin pathway in hepatocytes. The results of patients and cows with NASH and hepatocytes led us to speculate that defective protein expression of OXPHOS complexes may be an important factor in NEFA‐induced insulin resistance.

The mitochondrial electron transport chain mediated by OXPHOS complexes is the main source of cellular ROS.^40^ Defective protein expression of complexes (COI‐V) could induce a “leaky” transfer of electrons to molecular oxygen during OXPHOS, thus increasing ROS generation.^41^ Our study showed that patients and cows with NASH displayed severe oxidative stress. Importantly, the ROS content in the NEFA‐treated hepatocytes was significantly increased, which suggested that defects in OXPHOS complexes induce ROS overgeneration. It is well known that excessive ROS directly damages DNA, proteins and lipids and indirectly activates a variety of stress‐sensitive intracellular signalling pathways, such as p38MAPK and JNK, thus impairing the insulin pathway.^26^, ^42^, ^43^ In our study, NEFA treatment induced the overactivation of JNK and p38MAPK and inhibition of AKT in cow hepatocytes and HepG2 cells, and inhibition of JNK and p38MAPK could improve NEFA‐induced insulin resistance. Furthermore, antioxidant ALA treatment could inhibit the NEFA‐induced overactivation of JNK and p38MAPK and improve insulin resistance. These findings indicate that NEFAs induce overactivation of the ROS‐JNK/p38MAPK pathway, resulting in insulin resistance. However, ROS can be generated by mitochondria, endoplasmic reticulum stress and NADPH oxidase.^44^, ^45^ Therefore, it will be necessary to perform additional studies to investigate the source of ROS in NEFA‐treated hepatocytes.

Mitochondria biogenesis, respiration and oxidative phosphorylation are maintained by many molecular factors, such as PGC‐1α, Mfn‐2, NRF‐1 and TFAM.^46^, ^47^ Our data showed that the hepatic protein level of PGC‐1α was significantly decreased in patients and cows with NASH and in NEFA‐treated hepatocytes. Reduced PGC‐1α protein expression has been observed in the livers of patients with obesity, hepatic steatosis or NASH compared with healthy controls.^48^ In addition, previous studies have reported that low expression of PGC‐1α leads to defective mitochondrial OXPHOS, excessive ROS generation and insulin resistance.^15^, ^49^ Accordingly, these studies led us to speculate that PGC‐1α overexpression could reverse the lipotoxicity of NEFAs on hepatic mitochondrial dysfunction and insulin resistance. As expected, PGC‐1α overexpression enhanced COI‐V expression and restored membrane potential and ATP production in cow hepatocytes and HepG2 cells. Importantly, we found that PGC‐1α overexpression significantly decreased ROS content and inhibited the ROS‐JNK/p38MAPK pathway in cow hepatocytes and HepG2 cells, thus improving NEFA‐induced insulin resistance. Similarly, Wanagat et al also reported that PGC‐1α overexpression increased mitochondrial function, reduced hepatic TG content and improved insulin resistance in primary hepatocytes and Sprague‐Dawley rats,^20^ further confirming the beneficial role of PGC‐1α on mitochondrial dysfunction and insulin resistance in NASH. However, as shown in Figure 5B, NEFA treatment led to a striking increase in COII in the presence of Ad‐PGC‐1α in hepatocytes. We speculated that NEFA‐induced expression of COII in the presence of Ad‐PGC‐1α was a compensatory mechanism of mitochondria. Therefore, to investigate the effects of this compensatory mechanism, it will be necessary to perform additional studies.

Mfn‐2 is more abundant in the heart, skeletal muscle, brain and brown adipose tissue than in the liver cells. Its function in liver cells has usually been ignored in previous studies. In our study, we found that hepatic Mfn‐2 protein levels were significantly decreased in patients and cows with NASH and in NEFA‐treated hepatocytes. Importantly, Mfn‐2 overexpression significantly increased COI, COIII and COIV expression and ATP production, restored the membrane potential; suppressed the ROS‐JNK/p38MAPK pathway, thereby improving insulin resistance in hepatocytes. Furthermore, we also found that Mfn‐2 overexpression could reverse the inhibition of NEFAs on the expression of SSBP1 and POLG2 in hepatocytes. Our data demonstrated that Mfn‐2 overexpression could markedly decrease the lipotoxicity of NEFAs on mitochondrial function and insulin sensitivity, indicating that Mfn‐2 is a potential therapeutic target for insulin resistance in NASH. Previous studies showed that decreased Mfn‐2 expression caused an increase in ROS production in muscle cells, skeletal muscle and the liver.^50^, ^51^, ^52^ More importantly, our data demonstrated that Mfn‐2 overexpression significantly decreased ROS content. Overproduction of ROS can decrease the activities and expression of OXPHOS complexes.^53^ Therefore, we speculated that overexpression of Mfn‐2 decreased ROS content and rescued NEFA‐reduced protein expression of OXPHOS complexes in cow hepatocytes. However, the specific mechanism by which Mfn‐2 decreased ROS content needs to be investigated in future studies.

Non‐alcoholic steatohepatitis is an inflammatory disorder. Our data indicated that the blood concentrations of TNF‐α and IL‐6 were significantly increased in patients and dairy cows with NASH. In addition, overexpression of PGC‐1α or Mfn‐2 decreased NEFA‐induced TNF‐α and IL‐6 mRNA expression in cow hepatocytes and HepG2 cells. These data demonstrated that increased expression of PGC‐1α or Mfn‐2 might improve inflammatory disorders in patients or cows with NASH.

**Figure 9:**
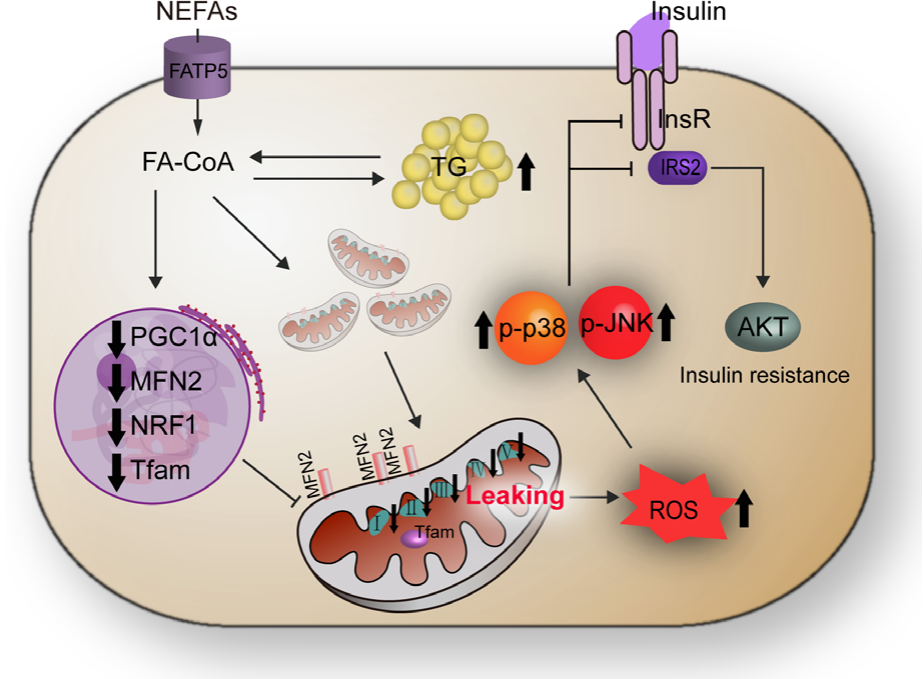
Proposed model of the mechanism that links mitochondrial dysfunction and insulin resistance in non‐alcoholic steatohepatitis. Patients or cows with NASH exhibited high blood concentrations of NEFAs, mitochondrial dysfunction and insulin resistance. Excessive NEFAs existed lipotoxicity and decreased the expression of mitochondrial function regulator PGC‐1α and Mfn‐2, OXPHOS complexes expression, ATP production and membrane potential, increased ROS content, thereby inducing mitochondrial dysfunction. Excessive ROS overactivated the hepatic JNK and p38MAPK pathway and then impaired insulin signalling pathway in patients or cows with NASH. FATP5, fatty Acid Transport Protein 5; FA‐CoA, fatty acyl‐CoA; InsR, insulin receptor

In summary, we revealed a mechanism of NEFA‐impaired mitochondrial function in the pathogenesis of hepatic insulin resistance in NASH (Figure 9). Patients or cows with NASH exhibited high blood concentrations of NEFA and insulin resistance. Excessive NEFAs increased ROS content and decreased expression of the mitochondrial function regulators PGC‐1α, Mfn‐2, NRF‐1, TFAM, OXPHOS complex expression, ATP production and membrane potential, thereby inducing mitochondrial dysfunction. Excessive ROS overactivated the JNK and p38MAPK pathways and induced insulin resistance in patients or cows with NASH. Importantly, PGC‐1α or Mfn‐2 overexpression reversed the mitochondrial dysfunction induced by NEFAs and improved insulin resistance in hepatocytes. Therefore, PGC‐1α and Mfn‐2 might serve as useful therapeutic targets for fatty liver in dairy cows and possibly in humans. Further prospective studies are needed to assess the mechanism of mitochondrial dysfunction involved in hepatic insulin resistance using hepatic samples from individuals with different degrees of pathological NAFLD, such as TG accumulation, steatohepatitis and liver cirrhosis. Furthermore, our data also demonstrated that cows with fatty liver may be ideal animal models for the study of human NAFLD.

## DISCLOSURE

No competing financial interests exist.

## AUTHOR CONTRIBUTIONS

W. W. G, X. L. D and L. L performed the experiments, prepared the figures and wrote the manuscript. X. W. L and G. W. L designed and supervised the study. H. Y. W and M. Z contributed to the collection of serum samples and the clinical analysis. Z. W performed the statistical analyses. X. B. L contributed to the editing of the manuscript. All authors saw and approved of the manuscript prior to submission.
