# Supplementary material for: NEFA‐induced ROS impaired insulin signalling through the JNK and p38MAPK pathways in non‐alcoholic steatohepatitis
Source: J Cell Mol Med. 2018 Mar 30;22(7):3408–22. doi: 10.1111/jcmm.13617 (PMC6010831; doi:10.1111/jcmm.13617)
Supplement: Supplementary file 1 [file JCMM-22-3408-s001.docx]

**Supplementary Data**

**Ethic Protocol**


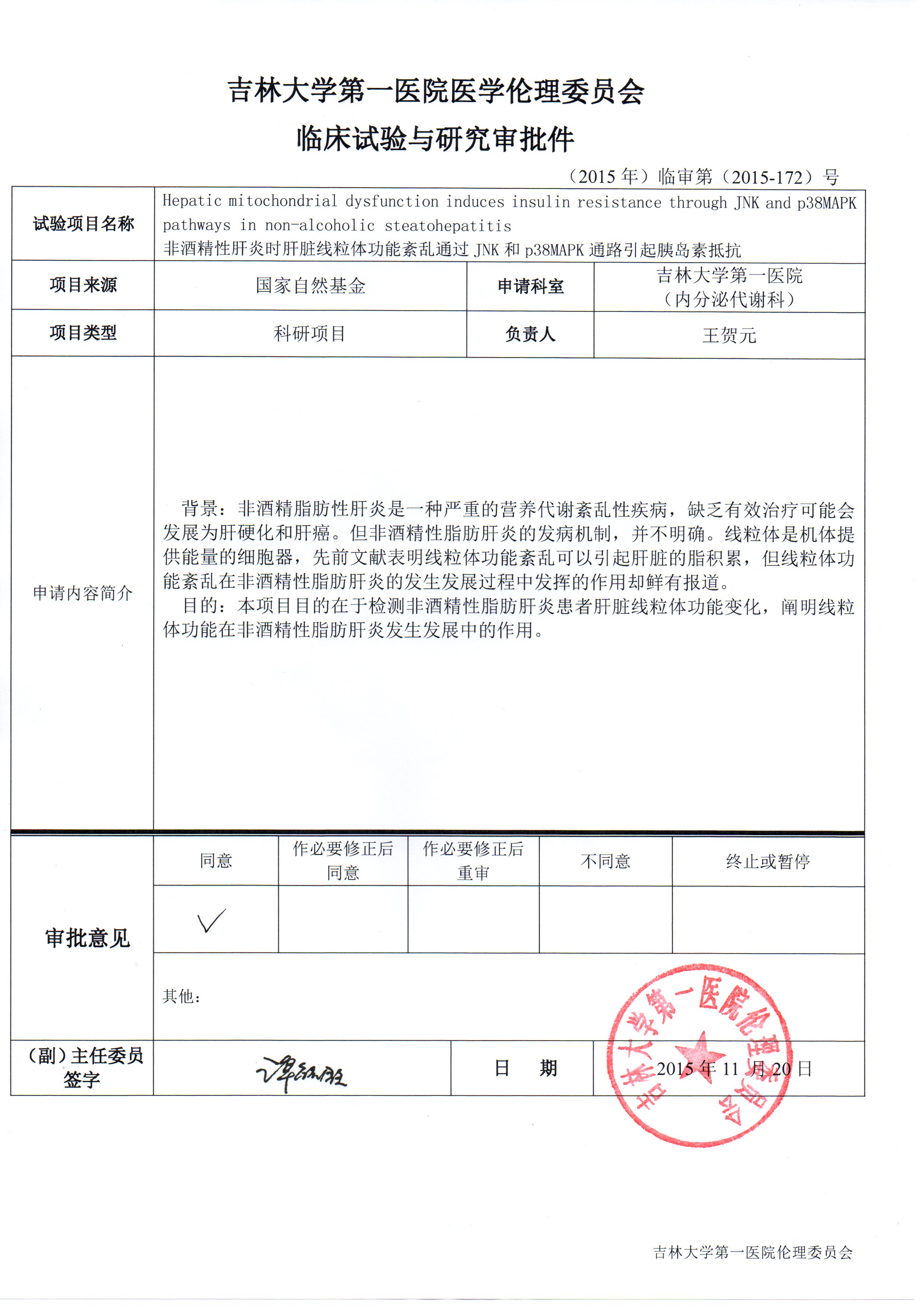

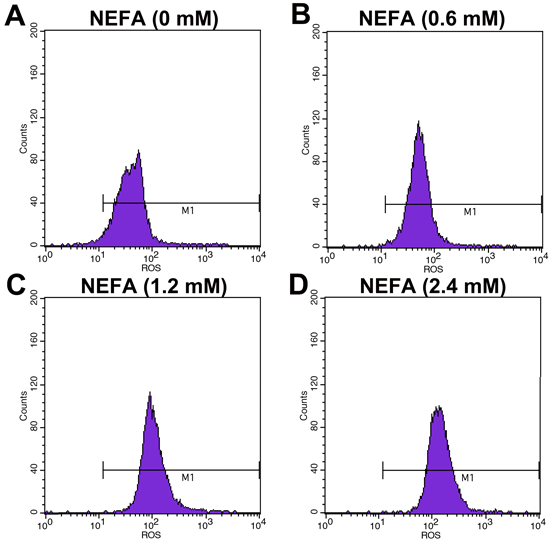


**Supplementary Fig. S1. The ROS content in cow hepatocytes.** Cow hepatocytes were treated with 0, 0.6, 1.2, and 2.4 *mM* NEFA, respectively. (A) The ROS content in the control cow hepatocytes. **(B)** The ROS content in the cow hepatocytes treated with 0.6 *mM* NEFA. **(C)** The ROS content in the cow hepatocytes treated with 1.2 *mM* NEFA. **(D)** The ROS content in the cow hepatocytes treated with 2.4 *mM* NEFA. Each treatment was repeated 8 times. ROS, reactive oxygen species; NEFA, non-esterified fatty acids.


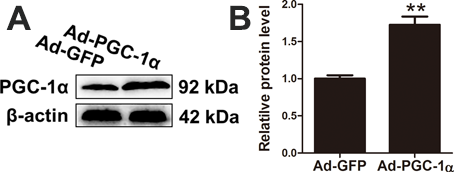


**Supplementary Fig. S2 Western blot analysis and quantification of PGC-1α in cow hepatocytes.**

Cells were infected with Ad-GFP or Ad-PGC-1α. (A and B) Western blot analysis and quantification of PGC-1α in cow hepatocytes. Data represent the mean ± SD. *p<0.05 and **p<0.01 compared with Ad-GFP group.


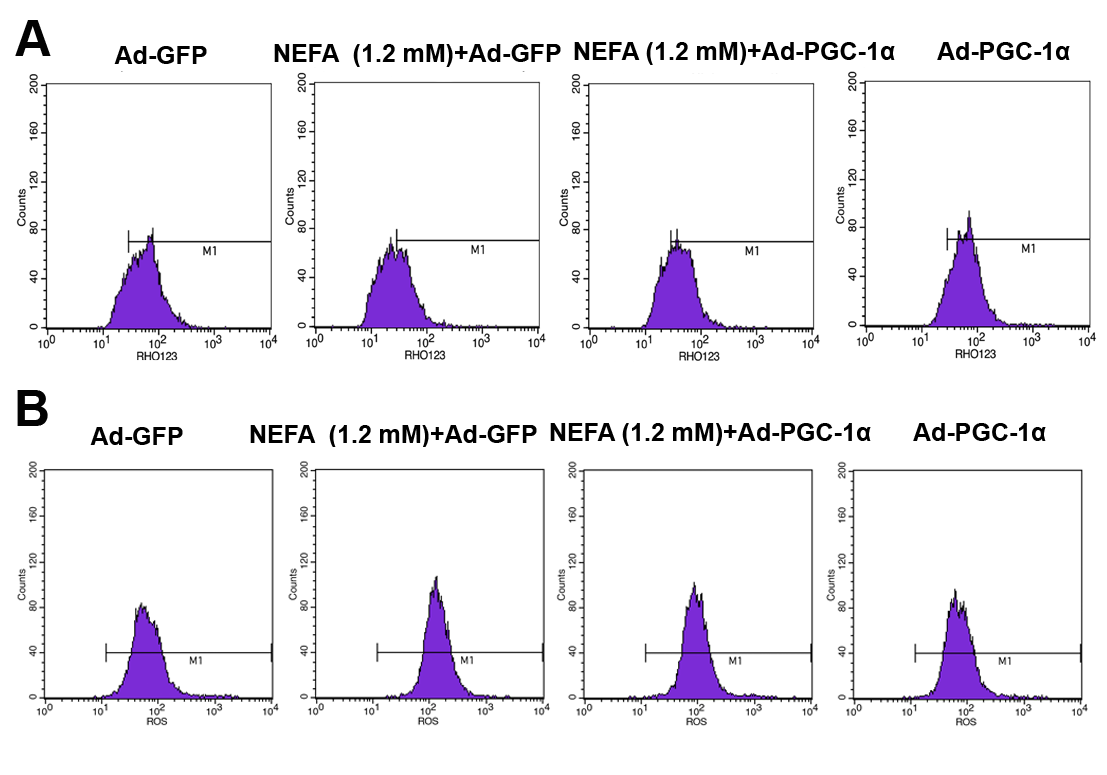


**Supplementary Fig. S3. The ROS content and** **membrane potential in cow hepatocytes.**

Cells were infected with Ad-GFP or Ad-PGC-1α and then treated with/without 1.2 *mM* NEFA, respectively.

(A) The membrane potential in the cow hepatocytes. Each treatment was repeated 8 times. **(B)** The ROS content in the control cow hepatocytes. ROS, reactive oxygen species; NEFA, non-esterified fatty acids.


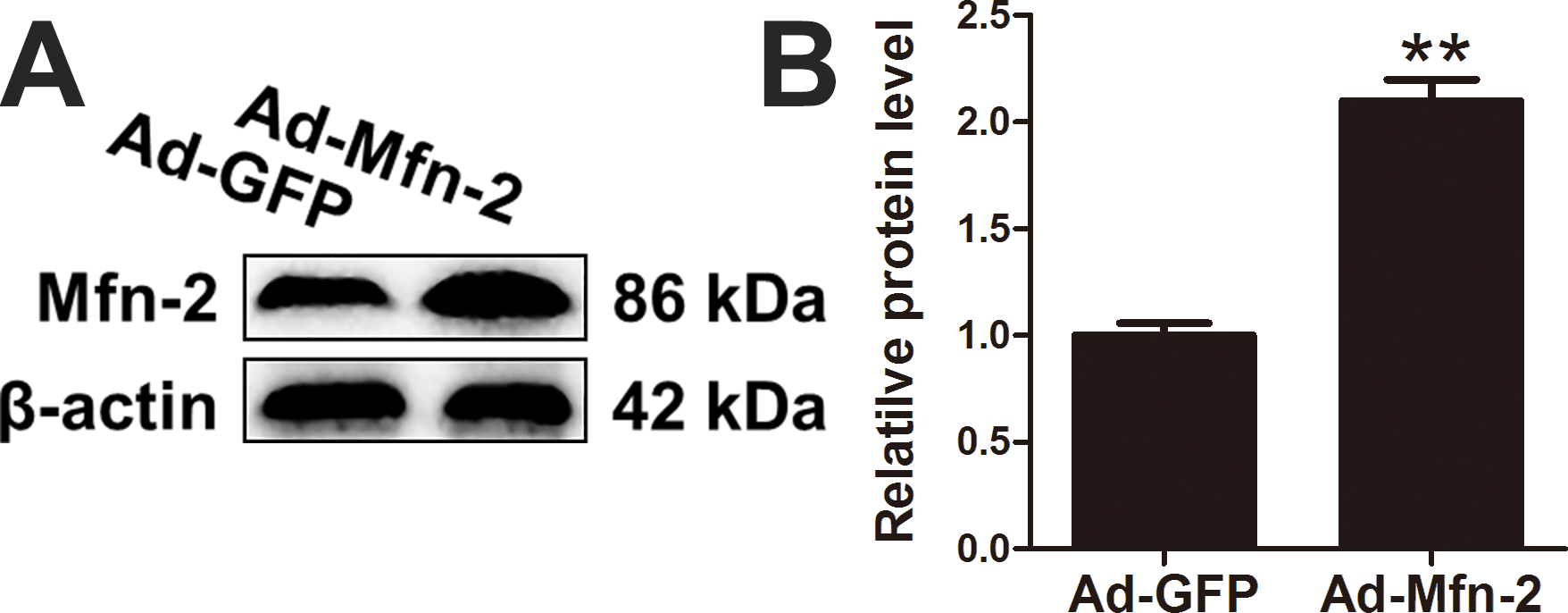


**Supplementary Fig. S4 Western blot analysis and quantification of Mfn-2 in cow hepatocytes.**

Cells were infected with Ad-GFP or Ad-Mfn-2. (A and B) Western blot analysis and quantification of Mfn-2 in cow hepatocytes. Data represent the mean ± SD. *p<0.05 and **p<0.01 compared with Ad-GFP group.


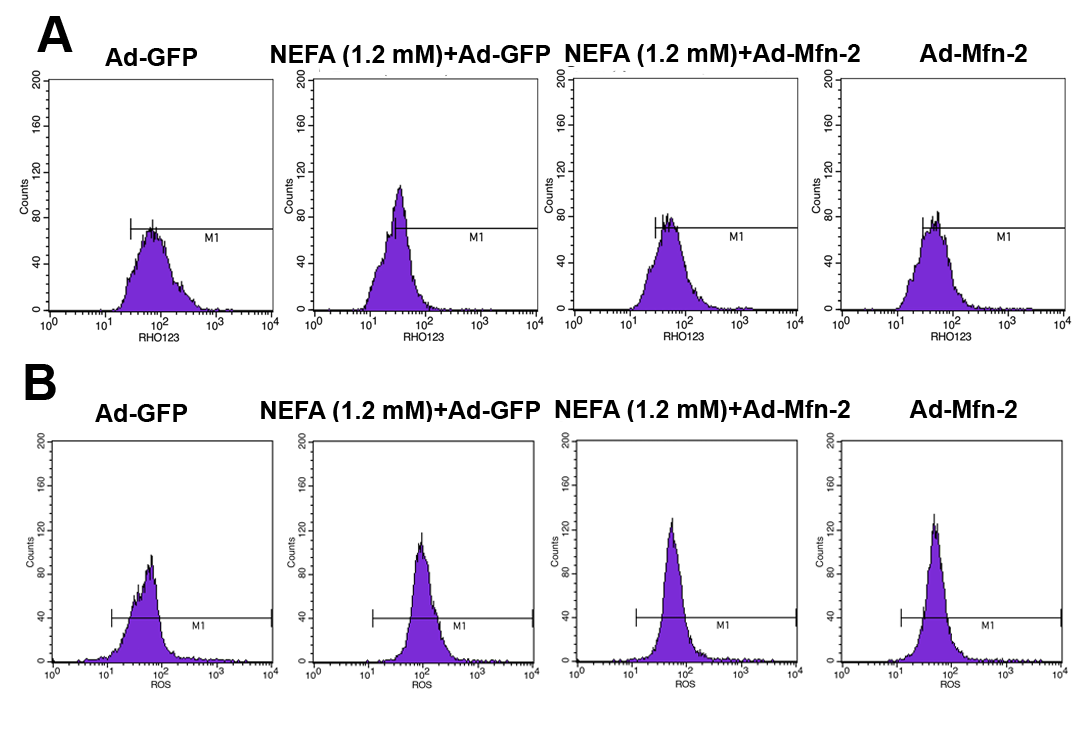


**Supplementary Fig. S5. The ROS content and** **membrane potential in cow hepatocytes.**

Cells were infected with Ad-GFP or Ad-Mfn-2 and then treated with/without 1.2 *mM* NEFA, respectively.

(A) The membrane potential in the cow hepatocytes. **(B)** The ROS content in the control cow hepatocytes.


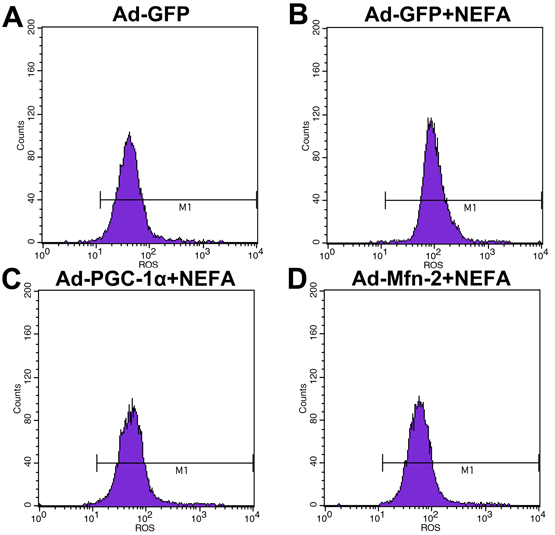


**Supplementary Fig. S6. The effects of overexpression of PGC-1α or Mfn-2 on the ROS content in HepG2 cells.** (A) The ROS content in Ad-GFP group. HepG2 cells were infected with Ad-GFP. (B) The ROS content in Ad-GFP+ NEFA group. HepG2 cells were infected with Ad-GFP and then treated with 0.8 *mM* NEFA. (C) The ROS content in Ad-PGC-1α+NEFA group. HepG2 cells were infected with Ad-PGC-1α and then treated with 0.8 *mM* NEFA. (D) The ROS content in Ad-Mfn-2+NEFA group. HepG2 cells were infected with Ad-Mfn-2 and then treated with 0.8 *mM* NEFA. Ad-GFP, green fluorescent protein adenoviral vectors; Ad-PGC-1α, PGC-1α adenoviral vectors; Ad-Mfn-2, Mfn-2 adenoviral vectors.
