# Supplementary material for: NEFA‐induced ROS impaired insulin signalling through the JNK and p38MAPK pathways in non‐alcoholic steatohepatitis
Source: J Cell Mol Med. 2018 Mar 30;22(7):3408–22. doi: 10.1111/jcmm.13617 (PMC6010831; doi:10.1111/jcmm.13617)
Supplement: Supplementary file 2 [file JCMM-22-3408-s002.docx]

**Supplementary Table S1. The baseline characteristics of controls and NASH subjects.**

|  | Normal subjects (n=6) | NASH subjects (n=8) |
| --- | --- | --- |
| Age | 43 ± 4 years | 46 ± 3 years |
| Females/males | 2/4 | 3/5 |
| BMI (kg/m^2^) | 22.3 ± 0.6 | 32.7 ± 1.3** |
| Fasting glucose (*mM*) | 5.1 ± 0.8 | 11.5 ± 3.6** |
| Fasting insulin (mU/L) | 4.7 ± 0.2 | 10.6 ± 0.6** |
| Fasting free fatty acids (*μM*) | 457 ± 38 | 816 ± 59** |
| Fasting triglycerides (mg/dl) | 86 ± 13 | 273 ± 68** |
| HbA1c (%) | 5.1 ± 0.3 | 7.5 ± 1.1** |
| HOMA-IR | 1.07 ± 0.3 | 5.43 ± 2.6** |
| IL-6 (ng/L) | 15.7 ± 2.8 | 38.2 ± 4.6** |
| TNF-а (ng/L) | 23.2 ± 4.7 | 64.3 ± 8.6** |
| ALT (U/L) | 27.6 ± 9.4 | 93.7 ± 20.3** |
| AST (U/L)  AST/ALT ratio | 25.2 ± 7. 7  0.95 ± 0.05 | 66.5 ± 18.3**  0.65 ± 0.11** |
| γ-GT | 23.2 ± 5.5 | 69.5 ± 13.2** |

**p*<0.05 and ***p*<0.01 compared with normal subjects. Values are mean ± SD. BMI, Body mass index; HbA1c, Hemoglobin A1c; HOMA-IR, homeostasis model assessment of insulin resistance; IL-6, interleukin 6; TNF-а, tumor necrosis factor-α; ALT, alanine aminotransferase; AST, aspartate transaminase; γ-GT, gamma-glutamyl transpeptidase; SD, standard deviation.

**Supplementary Table S2. The blood biochemical index of the control cows and cows with NASH.**

| Blood biochemical index | Control cows (n = 20) | NASH cows (n = 10) |
| --- | --- | --- |
| Body weight (kg) | 529.7 ± 15.1 | 541.5 ± 20.2 |
| Milk production (kg of milk/cow per day) | 28.1 ± 3.5 | 26.5 ± 4.5 |
| Glucose (*mM*) | 3.7 ± 0.33 | 4.5 ± 0.7** |
| Insulin (mU/L) | 15.2 ± 2.1 | 20 ± 3.3** |
| Free fatty acids (*μM*) | 281 ± 53 | 1296 ± 195** |
| IL-6 (ng/L) | 21.1 ± 2.5 | 62.6 ± 4.7** |
| TNF-а (ng/L) | 36.8 ± 6.9 | 118.2 ± 17.3** |
| ALT (U/L) | 18.2 ± 4.4 | 69.3 ± 17.6* |
| AST (U/L)  AST/ALT ration | 45.0 ± 9.6  2.6 ± 0.21 | 137.6 ± 25.4**  2.1 ± 0.13** |
| γ-GT | 22.5 ± 3.2 | 33.7 ± 5.4** |

**p*<0.05 and ***p*<0.01 compared with control cows. Values are mean ± SD. RQUICKI, Quantitative insulin sensitivity check index; IL-6, interleukin 6; TNF-а, tumor necrosis factor α; ALT, Alanine aminotransferase; AST, Aspartate transaminase; γ-GT, gamma-glutamyl transpeptidase.

**Supplementary Table S3. The primers sequences used for qRT-PCR.**

| Gene | Sequence number | Primer sequences (5’-3’) | | Length (bp) |
| --- | --- | --- | --- | --- |
| Bta-PGC-1α | NM_177945.3 | FOR CCCGTGCTACCTGAGAGAGA | 125 | |
|  |  | REV CTTGACTGGGATGACCGAAG |  |  |
| Bta-Mfn-2 | NM_001190270.1 | FOR TGGCGCAAGACTACAAACTG | 109 | |
|  |  | REV TCGTCCACCAACACAGAGAG |  |  |
| Bta-NRF1 | NM_001098002.2 | FOR CAGGGGTGGGCAATAAAAGA | 214 | |
|  |  | REV TTAGCAGGAAGTCAGAAAGG |  |  |
| Bta-TFAM | NM_001034016.2 | FOR TGGCACATCACAGGTAAAGC | 134 | |
|  |  | REV CCTCCCAAGATTTCATTTCG |  |  |
| Bta-COI | NM_205817.1 | FOR TATGGACTGGAACGGGAGAG | 162 | |
|  |  | REV GCTTCTTTGGACACTTGAGCA |  |  |
| Bta-COII | NM_174178.2 | FOR CAGAACCTGATGCTTTGTGC | 106 | |
|  |  | REV ACTCGTCAACCCTCTCCTTG |  |  |
| Bta-COIII | NM_174630.2 | FOR TCTCATTTGCTTCGTCTTGC | 197 | |
|  |  | REV TCTGGTGCTGTGGTGACATT |  |  |
| Bta-COIV | NM_001077831.2 | FOR ATCTCGGGTTTTTGGGTTGC | 334 | |
|  |  | REV GGGTGGTGGTCCAGGTTCTC |  |  |
| Bta-COV | NM_175796.3 | FOR TCCAGCCTGCCAGAGACTAT | 148 | |
|  |  | REV TGCACCTCCAGGGCATTTAG |  |  |
| Bta-POLG2 | NM_001075191.1 | FOR GTGGCTTGATTTCTGGTTACG | 160 | |
|  |  | REV CAGGGTTTCTATTGGCTCCTT |  |  |
| Bta-SSBP1  Bta-TNF-α  Bta-IL-6  Hsa-β-actin  Has-IL-6  Has-TNF-α | NM_001037466.1  NW-003104557.1  NW-00310889.1  NM_001101.4  XM_011515390.2  NM_000594.3 | FOR TCAGAGATGTGGCGTATCAGT | 175  234  144  403  261  106 | |
|  |  | REV CGTCCAGTCTACGGCTTCTC  FOR CTGCCGGACTACCTGGACTAT  REV CCTCACTTCCCTACATCCCTAA  FOR AACGAGTGGGTAAAGAACGC  REV CTGACCAGAGGAGGGAATGC  FOR CTAACAGTCCGCCTAGAAGCA  REV GTCATCACCATCGGCAATGAG  FOR GAACTCCTTCTCCACAAGCG  REV TTTTCTGCCAGTGCCTCTTT  FOR GGAGGGGTCTTCCAGCTGGAGA  REV CAATGATCCCAAAGTAGACCTGC |  |  |
